# Progress in Surgery of the Brain and Nerves

**Published:** 1904-02-06

**Authors:** 


### Progress in Surgery of the Brain and Nerves.

(*Concluded from page* 317.)

**Large Blood Cyst in the Arachnoid Space Simulating Brain Tumour.**—Taylor and Ballance [2] report this case. The patient was a man aged 37—family history of insanity, no history of syphilis. In February, 1902, he fell down, striking his head with some violence, and was unable to continue his work that day. Three months after the fall headaches commenced, which were relieved by drugs for three weeks. After this, medicinal treatment had no effect. The headache was a shooting pain referred to the right side. About five months from the date of injury he became drowsy and dull mentally, he was unable to walk without assistance, there being weakness, variable in degree, in the left arm and leg. Some blurring of the right optic disc was now noticed and he became noisy and lost control over his sphincters. He was considered to be insane. There was at this time increase of left knee-jerk and ankle clonus. He was admitted to the National Hospital on August 20th with a diagnosis of cerebral tumour probably of a fluid nature on account of the varying degree of the paralysis. On admission there was loss of memory for recent events, the gait was irregular and swaying, deep reflexes exaggerated on left side but no paralysis now, optic neuritis, most marked in right eye, horizontal nystagmus, intermittent headache. Operation was decided on and performed in three stages. First a large flap, including most of the anterior half of the scalp, was thrown down and the underlying bone removed with a saw. Five days later, in the second stage, the dura mater was opened, and there was then exposed a tense fluctuating and pulsating swelling of a maroon-red colour. It completely filled the wound and bulged through the opening in the skull. The pulsation was expansile like an aneurism. The opening in the skull was enlarged. Third stage, twelve days later, a temporary ligature was placed round the internal carotid artery and the wound again reopened, the opening in the bone measured $5\frac{1}{2}$ by $4\frac{1}{2}$ inches. The tumour, which was not adherent to the brain, was manipulated out of the cranial cavity with the fingers, it was attached by a slight adhesion to the dura posteriorly and to the cerebral arachnoid under the temporo-sphenoidal lobe. There were also other delicate connective threads passing between it and the cavity in which it lay. It measured 7 inches long, $4\frac{1}{2}$ inches from above downwards, and $1\frac{1}{2}$ inches in thickness. Its removal was attended by only slight bleeding. Eight months after the operation the patient was in perfect health.

**Cerebellar Tumours and their Treatment by Operation.**[3]—Professor Starr points out [4] that the operation for cerebellar tumours is attended with great difficulty and danger and that they are very often inaccessible to operation. The following two cases are reported by Hudson.[5] The first was a girl $9\frac{1}{2}$ years old, who developed headache, vertigo and vomiting, followed after some time by staggering gait and failing sight. The pupils were widely dilated and did not react to light. There was paralysis of the right facial nerve. The head was somewhat retracted with some rigidity of the neck muscles. Firm pressure over the right cerebellar fossa caused pain. Both knee-jerks were absent and there was weakness of the left arm and leg. Patient was unable to walk by herself, staggering and falling to the left if unsupported. Intense double optic neuritis, emaciation. Operation was decided on. On opening the right cerebellar fossa the dura bulged out tensely and on opening it a tumour was discovered, superficially "half as large as an English walnut." This was excised together with a small portion of brain around it. The patient recovered and grew cheerful, but the weakness and inco-ordination of movement persisted. A large hernia cerebri developed and a second operation was performed, a large hæmorrhagic cyst being found and evacuated deep in the right cerebellar lobe. Eight months after operation the patient was in a good state of health but was quite blind, the optic neuritis having gone on to atrophy. The second case was that of a man, aged 29, who at 12 years of age had been struck behind the right ear with a stone. A few years later he began to suffer from headaches and developed diplopia and a staggering gait; he also had attacks of vomiting. Failure of vision followed and epileptic attacks supervened. He was unable to walk or stand. There was also double optic neuritis, tenderness to pressure over the right occipital lobe, and paralysis of the right external rectus muscle and of the facial nerve. On opening the skull over the cerebellum and incising the tense dura mater a deep-seated tumour was found by palpation. It was about three-quarters of an inch below the surface of the brain, and when enucleated with the finger was found to be about the size of a hen's egg. Practically the whole right lobe of the cerebellum had been destroyed by the growth. The patient for the first few days appeared to be doing well and improving, when he contracted a severe form of diarrhœa and vomiting which killed him on the eleventh day after operation. The tumour was tuberculous.

**The Surgical Treatment of Facial Paralysis by Nerve Anastomosis.**—The *Medical Chronicle*[6] quotes from the "Annals of Surgery" a case reported by Cushing. The patient had received a bullet wound behind the right ear which had destroyed the lower part of the Fallopian aqueduct and facial nerve producing facial paralysis. Six weeks later, when the wound was healed, an incision was made along the anterior border of the sterno-mastoid and the spinal accessory nerve exposed at its entry into that muscle. The facial nerve was then exposed by incising the posterior border of the parotid gland. Both nerves being separated, the divided ends were approximated and sutured by fine silk over the posterior belly of the digastric. The incision in the parotid was sutured. After the operation the sterno-mastoid and trapezium were paralysed, and the shoulder drooped. On the 207th day after operation there was some co-ordination of the individual movements of expression; it was, however, impossible to elevate the shoulder or move the head to the left without calling the facial muscles into action. Closure of the eye was accomplished with less effort. An indirect response to faradism was observed for the first time. On the 287th day the electrical reactions were practically normal, individual co-ordinated movements were under greater control, the eye could be kept almost completely closed without effort. A violent elevation of the shoulder or movement of the head to the left still contracted the entire facial group of muscles. The writer remarks that the procedure is a delicate one and the success will depend upon (1) the care with which the nerves are handled; (2) the accurate approximation of the nerve stumps with least possible suturing and that placed in the sheath only; (3) absolute asepsis and hæmostasis; and (4) the least possible disturbance of the tissues lining the wound. Nicholl[7] relates the case of a woman on whom he operated for severe convulsive tic by dividing and suturing together the facial and hypoglossal nerves after removal of the posterior belly of the digastric. The results are not mentioned as time had not elapsed. He suggests that the distal end of the divided hypoglossal might be grafted on to the intact opposite hypoglossal.

**Nerve Suture and Nerve Regeneration.**—Henriksen[8] contributes two papers on this subject. In the first he commences by mentioning the different views held by various observers on nerve degeneration and regeneration, then relates a number of clinical cases to illustrate the conditions that take place after injury to peripheral nerves. The fact that sensibility often returns after union of nerves that have been long divided can only be explained on the supposition that conducting fibres have been formed in the peripheral part. The following questions are then put forward for consideration: (1) Can a severed nerve heal without degenerating by being sutured? (2) Can a degenerative nerve be supposed to be to some extent conducting? After a number of experiments on the peroneal nerve of the rabbit the former question is answered in the negative, the results being just the same on the peripheral end whether it was immediately sutured after section or not, viz., complete degeneration. The second paper deals mainly with the consideration of the second question. A further series of experiments were carried out, the nerve on one side simply being divided and on the other the peripheral end drawn out through the fascia to prevent possibility of union. The central and peripheral parts of the united and ununited nerves and the muscles were submitted to microscopic examination. The following are some of the conclusions arrived at:—When a nerve is divided it will lose its motor conductivity only after the lapse of some time—one to four days. Regeneration begins immediately after division, and takes place hand in hand with degeneration. The nerve nuclei within the myelin sheath proliferate, and after the lapse of a few days—seven—they are found to possess long processes which seem to be in connection with similar processes from other adjacent cells, and by and by they can be traced as continuous nerve fibres. Thus there cannot be any objection to the assumption that there may be a certain degree of sensory conduction during the whole process of regeneration. About the thirtieth day in the rabbit a myelin sheath is developed around the newly-formed fibres in the peripheral end. About the same time motor power is observed and increase in the weight of the muscles, and later on electrical reaction. The injured nerve is a bad conductor, and therefore the further from the periphery a nerve is hurt the longer time it will be until power is recovered. Although a divided nerve will unite equally rapidly whether it is sutured or not, still in all cases of injury operation should be undertaken in case there may be dislocation of the ends or infection of the wound. The time that may elapse before operation is done without risk of possibly incomplete recovery cannot be estimated at more than about a month. If sensation does not return or is incomplete at the time when scar tissue has been formed, complete recovery cannot be expected without operation. This development of sensation is important for diagnosis and prognosis. The injuries suffered by the muscle increase in proportion to the time that elapses before an interrupted nerve is sutured. Good results may, however, follow secondary suture carried out many years after the injury. Jessop relates a case where complete recovery took place after the lapse of nine years in a divided ulnar nerve. Both in primary and secondary suture it cannot be considered of any importance what suture material is used or how the suture is performed, the essential being that it should be done aseptically. In secondary suture it is of importance to remove all the scar tissue.

² Lancet, Aug. 29. ³ Ibid., Oct. 10. ⁴ Ibid., Aug. 15. ⁵ Amer. Jour. Med. Scien., September. ⁶ Med. Chron., July. ⁷ Lancet, Oct. 3. ⁸ Ibid., April 11 and April 18.

